# The evolving picture of the glioblastoma genome

**DOI:** 10.18632/oncotarget.133

**Published:** 2010-08-04

**Authors:** Donald W. Parsons

**Affiliations:** Texas Children’s Cancer Center, Baylor College of Medicine, Departments of Pediatrics and Molecular and Human Genetics, Houston, TX 77030, USA

Survival rates for patients with glioblastoma multiforme (GBM), the most common malignant brain tumor, remain dismal despite decades of basic and clinical research. New treatment strategies are desperately needed. Consequently, the high-throughput genomic technologies developed to facilitate examination of cancer genomes have recently been focused on GBM, with goals of identifying novel molecular targets and developing a clinically-relevant biological classification of this deadly cancer.

These genomic analyses have provided an unprecedented view of the complex genetic landscape of GBM, confirming the central role of both well-established and previously-unsuspected genes in the development of these tumors [1,2]. In addition, despite the plethora of genes found to be altered in GBMs, these data have allowed a semblance of order to be applied to these tumors through the identification of a small number of core pathways critical to GBM pathogenesis and the elucidation of discrete molecular subgroups of GBM [3]. The list of biologically-relevant GBM genes remains incomplete, however, due in large part to the logistical challenge of establishing the functional significance of the numerous genes found to be infrequently altered (between 1 and 5%) in these cancers.

The detection of focal regions of recurrent copy number gain or loss (amplifications or homozygous deletions) can be a useful tool for the identification of candidate cancer genes. Using such a high-resolution genomic approach, Duncan et al. have identified two genes as potentially relevant to the emerging picture of the GBM genome, *ERRFI1* (located at chr1p36) and *TACC3* (located at chr4p16) [4]. Genomic alterations in these two regions have been reported in a number of human cancers, but the specific target genes of these copy number alterations are not known. The Digital Karotyping and SNP array data provided by Duncan et al. have provided initial evidence for two reasonable candidates. Importantly, given the significant challenge of distinguishing between altered genes which are drivers (i.e. genes that contribute to tumorigenesis) and those that are merely passengers (i.e. genes that accumulate alterations during tumor development but are not biologically relevant), preliminary studies of the potential functional significance of the candidate genes have been conducted.

Recurrent focal deletions at 1p36, previously reported in numerous cancers including GBM, were found by Duncan et al. in their cohort of GBMs and validated using data from The Cancer Genome Atlas (TCGA) project. Two distinct minimal deleted regions were identified, with the most commonly-deleted region containing a single gene, *ERRFI1*. *ERRFI1* expression was also found to be downregulated in ~34% of GBM samples analyzed, and functional studies of a cell line with a homozygous deletion of *ERRFI1* revealed impaired wound-healing and a lower rate of trans-well migration after transfection with a vector containing *ERRFI1*, suggesting *ERRFI1* as a potential tumor suppressor gene in GBMs. Similarly, focal gains at 4p16, most commonly involving *TACC3*, and overexpression of *TACC3* were found in tumor samples, raising the possibility of *TACC3* as a potential GBM oncogene.

Both of these genes are biologically-plausible as GBM-related candidates. *ERRFI1* acts as a negative regulator of EGFR and the ERbB family, while dysregulation of TACC (transforming acidic coiled coil) genes, components of the centrosome, has been implicated in cancer development. Significant work remains, however, in order to confirm the relevance of these genes for the pathogenesis of GBM and identify them as potential targets of molecular therapeutics. In particular, additional functional data firmly establishing the genes as “drivers” and demonstrating their specific biological role in GBM development are required. In addition, clarification of how these genomic data fit in with the evolving picture of the molecular classification of GBM will be illuminating: for example, are alterations of *ERRFI1* specifically identified in the subgroup of GBMs with downstream evidence of *EGFR* activation that lack *EGFR* amplification or mutation [3]? Further research building on the foundation of this intriguing genomic data will surely add to our evolving picture of the glioblastoma genome. Hopefully it will also contribute to the improved care of our patients with these deadly cancers.

## References

[1] McLendon R, Friedman A, Bigner D, Van Meir EG, Brat DJ, Mastrogianakis M, Olson JJ, Mikkelsen T, Lehman N, Aldape K, Alfred Yung WK, Bogler O, Vandenberg S, Berger M, Prados M, Muzny D (2008). Comprehensive genomic characterization defines human glioblastoma genes and core pathways. Nature.

[2] Parsons DW, Jones S, Zhang X, Lin JC, Leary RJ, Angenendt P, Mankoo P, Carter H, Siu IM, Gallia GL, Olivi A, McLendon R, Rasheed BA, Keir S, Nikolskaya T, Nikolsky Y (2008). An Integrated Genomic Analysis of Human Glioblastoma Multiforme. Science.

[3] Verhaak RG, Hoadley KA, Purdom E, Wang V, Qi Y, Wilkerson MD, Miller CR, Ding L, Golub T, Mesirov JP, Alexe G, Lawrence M, O’Kelly M, Tamayo P, Weir BA, Gabriel S Integrated genomic analysis identifies clinically relevant subtypes of glioblastoma characterized by abnormalities in PDGFRA, IDH1, EGFR, and NF1. Cancer Cell.

[4] Duncan CG, Killela PJ, Payne CA, Lampson B, Chen WC, Liu J, Solomon D, Waldman T, Towers AJ, Gregory SG, McDonald KL, McLendon, Bigner, Yan H (2010). Integrated genomic analyses identify ERRFI1 and TACC3 as glioblastoma-targeted genes. Oncotarget.

